# The impact of digital educational games on student’s motivation for learning: The mediating effect of learning engagement and the moderating effect of the digital environment

**DOI:** 10.1371/journal.pone.0294350

**Published:** 2024-01-11

**Authors:** Youling Li, Di Chen, Xinxia Deng

**Affiliations:** 1 Graduate School, Rattana Bundit University, Bangkok, Thailand; 2 Research Center for Collaborative Innovation of Airport Economy, Guangdong University of Foreign Studies South China Business College, Guangzhou, Guangdong, China; 3 Faculty of Arts and Design, Guangdong Baiyun University, Guangzhou, Guangdong, China; University of Granada: Universidad de Granada, SPAIN

## Abstract

The utilization of digital educational infrastructure in schools has propelled digital educational games to the forefront of educational innovation. Despite an abundance of empirical studies on the relationship between digital educational games and student’s motivation for learning, a consensus has yet to be reached. This study aims to bridge existing research gaps by adopting a mixed-methods approach grounded in behaviorist learning theory and contextual cognitive theory. A detailed questionnaire was disseminated to students from three distinct university in Thailand. After the exclusion of invalid responses, a robust sample of 434 valid responses was curated and utilized for analysis. Utilizing SPSS and MPLUS software, empirical analyses were conducted to explore the impact of digital educational games on student’s motivation for learning. Research results indicate that: First, digital educational games positively influence student’s motivation for learning; Second, learning engagement serves as a mediator between digital educational games and student’s motivation for learning; Third, the digital environment moderates the relationship between digital educational games and student’s learning engagement. Notably, the positive impact of digital educational games on student learning engagement is amplified in a more immersive digital environment. This study contributes to behaviorist theory and social cognition theory by elucidating how digital educational games affect student’s motivation for learning through their engagement and by highlighting the moderating role of the digital environment. Practically, these findings underscore the significance of digital educational games and the digital environments in schools to enhance student’s motivation for learning.

## 1. Introduction

Education is pivotal in social development and individual growth [1]. As information technology advances, digital educational games have emerged as a novel and noteworthy tool for academic innovation, garnering increasing societal attention and exploration [2]. These games, based on computer and Internet technologies, are highly favored by educators and students alike due to their unique learning methods and interactivity. In contrast to traditional education approaches, digital educational games offer learners a fresh and distinctive learning experience, presenting educational content in a gamified format that simulates real-life scenarios, thus creating a captivating and interactive learning environment. These games find extensive application across various subject areas, encompassing language learning, mathematics, science, humanities, and social sciences. They serve as valuable supplements to classroom teaching and assume a vital role in diverse educational settings such as independent learning, supplementary teaching, and personalized education [3].

The educational value and potential of digital educational games have attracted tremendous scholarly attention. Irina (2023) conducted in-depth studies on the impact of these games on students’ motivation for learning to show their efficacy in the field of education [4]. Student’s motivation for learning is a crucial aspect of the educational process, influencing their commitment to learning tasks and their perception of the learning experience. As an emerging educational tool, digital educational games have the potential to enhance student’s motivation for learning [5]. However, the influencing mechanism involved in the impact of these games on student’s motivation for learning is yet to be identified. Therefore, this study aims to examine the impact of digital educational games on student’s motivation for learning, explaining this impact through the mediating role of learning engagement and the moderating role of the digital environment. Student’s learning engagement refers to students’ subjective perception of their experience, involvement, and engagement in the game process, while the digital environment encompasses the digital educational resources, technical equipment, and teaching support provided by schools. This study aims to identify the mechanisms by which digital educational games affect student’s motivation for learning by analyzing the mediating effect of learning engagement and the moderating effect of the school’s digital environment.

To investigate the impact of digital educational games on student’s motivation for learning, this study combines social cognition theory and behaviorist theory [6]. Social cognition theory explains how learning environments can be contextualized and realistic scenarios simulated to stimulate and motivate students. By providing a contextualized learning environment, digital educational games help students better understand and master knowledge, resulting in a livelier and more exciting learning process. Furthermore, these games enable students to experience the learning content through realistic scenario simulations, stimulating their interest and motivation to learn. The study concludes that digital educational games can enhance students’ motivation by offering a contextualized learning environment. On the other hand, behaviorist theory elucidates how behavioral outcomes and reward mechanisms influence students’ motivation for learning. In digital educational games, rewards help students better comprehend and master knowledge while motivating them to continue learning and exploring. Through reward mechanisms, digital educational games actively engage students in the learning process. Therefore, this study argues that reward mechanisms in digital educational games can further enhance student’s motivation for learning.

In summary, social cognition theory emphasizes the influence of the learning environment on the learning process and outcomes [7]. In digital educational games, students are immersed in a contextualized learning environment that stimulates and motivates them through the simulation of real-life scenarios and situations. The game’s tasks, challenges, and interactions allow students to explore new knowledge and solve problems, resulting in a deeper learning experience [8]. Social cognition theory provides a theoretical framework for understanding how digital educational games enhance student motivation by creating engaging learning environments. Conversely, behaviorist theory focuses on learning outcomes and reward mechanisms for learning behavior [9]. In digital educational games, students receive immediate rewards and a sense of achievement for their participation and completion of tasks, such as earning points, unlocking new levels, or receiving virtual incentives. This reward mechanism stimulates students’ intrinsic motivation to actively engage in learning activities and achieve better learning outcomes [10]. Behaviorist theory provides the theoretical basis for understanding how digital educational games enhance students’ motivation through reward mechanisms.

**Fig 1:**
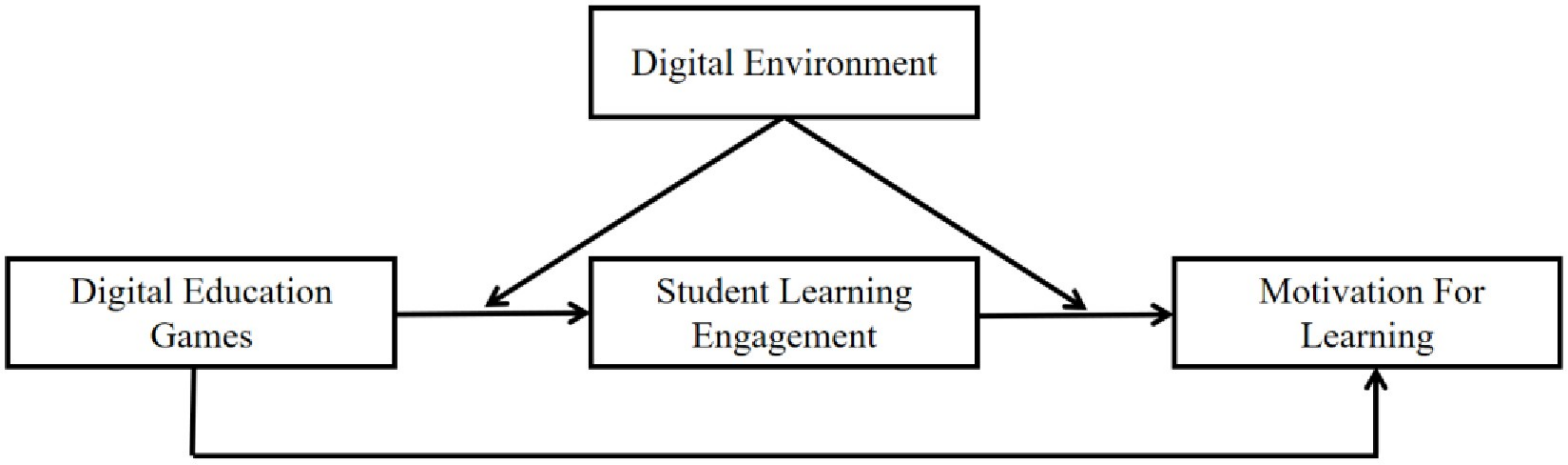
Proposed moderated mediation model.

Based on social cognition and behaviorist theories, this study proposes a mediated moderation model. The study argues that student learning engagement which refers to students’ subjective perception of their involvement in digital educational games, including their interest, engagement, and participation in the games plays a vital mediating role in the impact of digital educational games on student’s motivation for learning [11]. Through the mediation of learning engagement, positive experiences and learning outcomes in games can translate into positive attitudes and behaviors toward learning. Additionally, the model considers the digital environment as a moderator. The digital environment, comprising teaching and learning facilities, technological equipment, and support resources, plays a crucial role in effectively implementing digital educational games and fostering student learning engagement [12]. The quality of the digital environment may influence students’ engagement with digital educational games, thereby affecting their motivation for learning. To provide a clearer representation of the mediated moderation model, Fig 1 illustrates the relationships between the hypotheses proposed in this study, the mediating role of student learning engagement, and the moderating role of the digital environment at school.

## 2. Literature review and theoretical foundations

### 2.1. Digital educational games and students’ motivation for learning

In today’s digital era, digital educational games have gained significant attention within the realm of education. Although the term "digital games" is universally acknowledged, its implications and relevance in the educational context differ among cultures and countries [13]. For clarity in this study, we define "digital educational games" as interactive activities, facilitated by electronic devices, designed with an educational purpose. Such games are crafted to enhance students’ understanding and command over complex concepts and knowledge. For example, "Kerbal Space Program" serves as an aerospace simulation, aiding engineering and physics students in navigating intricate aerospace principles [14]. Digital educational games are not merely pedagogical instruments. A plethora of modern games mirror real-world situations, dilemmas, and environments, presenting students with a dynamic learning platform [15]. These games can cultivate diverse skills, such as collaboration, analytical thinking, and problem-solving. "SimCity," a simulation focused on urban planning and management, equips students in the field with insights into the complexities of urban evolution [16]. To ensure the comprehensive relevance and accuracy of our research, we’ve considered the unique contexts of various countries and cultures, particularly during the phases of questionnaire formulation and dissemination, affirming the research’s credibility and pertinence.

According to behaviorist theory, students’ responses are influenced by external stimuli, such as classroom activities [17]. Behaviorist theory, also referred to as the "stimulus-response" theory, is applied in digital educational games. These games provide students with various learning scenarios of differing difficulty levels, gradually introducing clues from simple to complex. Feedback in the form of scores acts as a stimulus, motivating students to achieve the desired response as set by the teacher [18]. Through repetitive practice and reinforcement, teachers employ digital games to help students establish the correct stimulus-response connection, while increased student motivation signifies the accomplishment of the game’s objectives [19]. Digital educational games serve as innovative teaching tools that significantly enhance student’s motivation for learning. A study conducted with secondary school students demonstrated that those who utilized digital educational games exhibited greater motivation, initiative, focus, and willingness to explore compared to students in traditional teaching settings (Anja Hawlitschek, (2017) [20]. The interactive and challenging nature of these games fosters students’ engagement, encouraging them to invest time and effort in problem-solving and task completion [21]. Immediate feedback and incentives provided by digital educational games further contribute to heightened student motivation. By structuring learning tasks as games, with chapter-specific trivia points as vital clues for progression, students earn points within designated time limits. Faster completion times yield higher point rewards, and accumulated points can be exchanged for specific bonuses, thereby motivating students to take initiative in their learning. Moreover, the digital game system recognizes outstanding performance by displaying the names of high-achieving students, fostering a sense of accomplishment and encouraging positive behaviors, such as attentive listening in class [18]. This positive learning experience cultivates sustained motivation and an active approach to learning. Additionally, digital educational games facilitate personalized learning experiences that cater to students’ individual levels and needs. This tailored approach stimulates students’ interest and motivation, leading to greater involvement in their education [18, 22]. Research indicates that personalized digital educational games improve students’ learning outcomes, satisfaction, and overall enjoyment of the learning process. By effectively motivating students during teaching, digital educational games enable teachers to facilitate strong engagement. Based on these insights, we propose the following hypothesis:

Hypothesis 1: There is a positive relationship between digital educational games and students’ motivation for learning.

### 2.2. The mediating role of student learning engagement

The social cognition theory highlights the impact of environmental factors on mental activities during the learning process. Digital educational games play a crucial role in creating a conducive learning context for students and enhancing their learning engagement. There are four key aspects to consider regarding the impact of digital educational games on learning engagement.

First, context creation: Digital educational games provide a virtual environment that resembles the experience of playing an online game. This virtual context stimulates students’ interest in learning and enhances their engagement with the learning tasks. The interactive and immersive features of these games contribute to students’ perception and engagement [23, 24].

Second, interactivity: Digital educational games incorporate various interactive elements, including role-playing, problem-solving, and teamwork. Students can actively engage with the game and collaborate to complete tasks. This sense of interactive engagement effectively increases their perceived level of engagement.

Third, instant feedback: Similar to online games, digital educational games offer instant feedback mechanisms that provide feedback based on student behavior and performance. This feedback allows students to gain a clear understanding of their progress and encourages them to continually improve their learning strategies.

Last, emotional experience: Digital educational games often evoke emotional responses through elements such as sound effects, images, and storylines [25]. Students can derive positive emotional experiences, such as a sense of achievement, self-confidence, and enjoyment, while playing these games. These emotional experiences contribute to their perception of active participation in the learning process. Based on these observations, we propose hypothesis 2:

Hypothesis 2: A positive correlation exists between digital educational games and students’ learning engagement.

According to behaviorist Theory, students will respond correspondingly to the external stimuli they receive. Student learning engagement refers to students’ subjective feelings and perceptions about their learning activities and environment, encompassing their interest in the learning task, commitment to the learning process, and overall satisfaction with the learning environment [23]. Research has demonstrated that students exhibit higher levels of engagement when they perceive learning activities to be meaningful and relevant [23]. For instance, Stipek’s (2002) study revealed a positive correlation between students’ learning engagement in tasks and their motivation to learn. When students perceive that the learning task aligns with their interests and needs, they are more motivated to participate in learning [26]. Additionally, perceptions of student engagement stimulate curiosity and enthusiasm about learning (Fredricks et al., 2004) [27]. Perceived student engagement significantly enhances students’ motivation to learn. In summary, we propose Hypothesis 3:

Hypothesis 3: Student learning engagement is positively related to students’ motivation for learning.

In summary, digital educational games have the potential to enhance learning engagement by providing an interactive and dynamic learning environment with instant feedback mechanisms similar to those found in online games. Student’s learning engagement, which encompasses students’ perceptions and experiences of the learning environment and activities, affects their feelings toward digital educational games, consequently impacting their motivation for learning [26]. Learning engagement, therefore, could mediate the relationship between digital educational games and student’s motivation for learning. Previous research has highlighted the significance of student learning engagement in relation to career development. For instance, Panigrahi’s (2021) study revealed that student learning engagement mediated the relationship between the online learning environment and learning performance [28]. Additionally, according to Guo Jianpeng and Guogun Ji (2019), student learning engagement act as mediators between university students’ learning experiences and learning outcomes [29]. In summary, digital educational games enhance student learning engagement, leading to increased motivation to learn. Therefore, we propose:

Hypothesis 4: Student learning engagement mediates between digital educational games and student motivation for learning.

### 2.3. The moderating role of the digital environment

According to the social cognition theory, the learning environment exerts an influence on learning processes and outcomes, shaping students’ learning behaviors and achievements, which can be influenced by external factors [30]. The school’s digital environment serves as a crucial resource for students, providing them with the necessary technological facilities and resources for digital educational games, such as computers, campus-wide WiFi connectivity, digital learning platforms, and more. These resources enable students to easily access and utilize information, social networks, and various forms of support related to digital educational games, all of which play a vital role in the students’ learning process. Schools with excellent digital infrastructure are better equipped to implement digital educational games compared to schools lacking sufficient digital infrastructure and having a lower level of digital environment [31]. In other words, the digital environment can mediate the mechanisms through which digital educational games impact student’s learning engagement.

Specifically, when examining the factors influencing the digital environment, it’s essential to recognize that the digital environment encompasses not only the quality of technological devices and network connections but also the digital literacy of both teachers and students [32]. Specifically, the digital environment includes the availability of digital devices, the quality of network connections, and the digital literacy of teachers and students. These elements can influence students’ exposure to and utilization of digital educational games, subsequently affecting their engagement perception [33–35].

Student digital literacy plays a pivotal role here. Digital literacy goes beyond merely using technological tools [36]; it’s about how students interact with digital content effectively, safely, and critically. Students with high digital literacy can not only operate technology proficiently but also evaluate, analyze, and create digital information [37]. Moreover, students’ access to digital devices is a determining factor, indicating whether they can easily obtain and use computers, tablets, or smartphones at school, home, or elsewhere [38]. Without appropriate device access, students’ experiences and opportunities in using digital tools for learning might be compromised [39].

Furthermore, a school’s digital infrastructure offers students a robust platform [40], while teachers’ digital literacy aids in handling challenging tasks, providing feedback, and offering professional growth advice [41]. Students in schools with a more advanced digital environment are more likely to recognize and perceive the innovative methods of teaching and learning through digital educational games [42]. However, deficiencies in the school’s digital environment can adversely affect the relationship between digital educational games and student learning engagement. For instance, unstable internet connections or insufficient digital devices can hinder students from immersing themselves fully in the context of digital educational games, reducing their engagement perception [43]. Additionally, if teachers lack digital literacy, leading to inadequate guidance on digital educational games, students might feel confused and demotivated, affecting their engagement with these games [44]. If families cannot provide digital devices for their children, the school’s digital infrastructure should offer proper counseling, equipment support, and assistance, helping students overcome low self-esteem caused by familial circumstances, allowing them to re-engage in their studies with a more positive mindset. A school with a superior digital environment is expected to deliver an enhanced digital educational gaming experience, leading to heightened student learning engagement. Therefore, we propose the following hypothesis:

Hypothesis 5: The digital environment moderates the relationship between digital educational games and student’s learning engagement. Specifically, the higher the level of the digital environment, the stronger the effect of digital educational games on student’s learning engagement, while the weaker the opposite effect.

The digital environment is a controllable space shaped by intelligent algorithms processing various information. It leverages network technology’s immediate, practical, and convenient aspects to provide individuals with specific digital experiences and emotions during information transmission, collection, analysis, and integration. In the context of digital educational games, the digital environment creates an optimal learning space for learners with its distinctive characteristics, mediating students’ learning engagement and motivation for learning. This mediation is demonstrated as follows:

Firstly, the digital education environment facilitates personalized learning for students. De Benito (2020) asserts that digital environments are fundamental in modern education trends, enabling autonomous and customized learning focusing on the student. Students’ information behaviors in digital environments indicate their participation in diverse educational activities contributing to personalized learning [45]. McNaughton’s (2018) study shows that students in digital environments utilize personal devices to access technology, set their goals, access required resources, and control their learning pace, thus enhancing their personal digital educational gaming experience and actively engaging in classroom learning [46]. Therefore, improving digital environments during digital educational games aids students in tailoring their learning experiences and promoting active learning.

Secondly, digital environments enhance students’ learning engagement through gamification elements. Gamification is widely adopted in educational settings, as evidenced by Barata (2015) and Nand (2019), who have used game design elements to heighten student engagement and motivation [47, 48]. Past research extensively discusses various dimensions of digital games and their significance in gaming experiences, such as narrative elements in gamification [48]. Alexiou’s (2018) study demonstrates that narrative elements foster users’ emotional engagement through empathy and identification with game characters, thereby promoting cognitive engagement and intrinsic motivation for learning [49].

Thirdly, digital environments offer diverse learning resources. According to Noskova (2021), digital learning environments encompass social experiences, scientific knowledge, and educational resources [50]. In digital educational games, students gain access to a wealth of learning resources through digital environments, identify new structures of learning content, achieve novel learning outcomes within the digital educational environment’s help, and bolster their learning motivation.

In conclusion, digital educational games are facilitated by improving the digital environment, which promotes students’ personalized learning experiences, enhances their perception of participation through gamification elements, and provides diverse learning resources. This journey within the digital environment aids students in constructing their learning communities, fostering a sense of belonging and motivation to learn. Therefore, we propose the following hypotheses:

Hypothesis 6: The school’s digital environment can regulate the relationship between students’ learning engagement and students motivation for learning. The higher the level of the school’s digital environment, the stronger the effect of student learning engagement on student motivation for learning, and vice versa.

Overall, a higher level of the digital environment provides support in terms of digital devices, digital platforms, and instructional resources for student learning, facilitating a quick adaptation and identification with the teaching and learning approach of digital educational games. Teachers’ digital literacy plays a crucial role in motivating career-ready students by offering resources, support, and feedback, helping them acquire learning skills, confidence, and a sense of belonging through digital educational games. Considering the mediated relationship (H4) and the moderated relationship (H5) established in the study, we propose a model of moderated mediation. This model suggests that the indirect effect of digital educational games on student motivation, through the perception of student learning engagement, depends on the level of the digital environment. Specifically, digital educational games can enhance the relationship between digital educational games and student learning engagement, subsequently reinforcing the mediating role of student learning engagement in influencing the relationship between digital educational games and student’s motivation for learning. Based on this, we propose the following hypothesis:

Hypothesis 7: The school digital environment may positively modulate the mediating role of students’ learning engagement in the relationship between digital educational games and students’ motivation for learning. In other words, at a high level of the school digital environment, students participating in digital educational games will perceive more robust engagement and be more motivated for learning.

## 3. Research methods

### 3.1. Sample and procedure

Data for this study were collected through a questionnaire survey targeting the Thai education industry to evaluate the proposed model. The three universities in Thailand, from which students were selected as the research population using a random sampling method, have a combined student body of approximately 45,000. To ensure the convenience, applicability, and validity of the sample size, this research focused on these universities.

Initially, the researchers leveraged the personal contacts of the MA in Educational Management (EMD) students to establish connections with the school authorities. The study objectives were then thoroughly communicated via WhatsApp and email, emphasizing that the survey was for academic purposes and would have no adverse impact on the students or the school. In this investigation, data were amassed from participants utilizing a questionnaire survey. Prior to the initiation of the study, the purpose and procedure of the research were elucidated to all participants, alongside their rights, accompanied by the presentation of an informed consent form. Upon securing their verbal informed consent, participants proceeded to complete the questionnaire. All survey responses were anonymized to safeguard the privacy of the participants, thus precluding any disclosure of their identities. This study has been reviewed and approved by Ethics Committee of Rattana Bundit University.

Data collection involved the use of both online and paper questionnaires. A total of 535 questionnaires were distributed, and 450 questionnaires were returned, resulting in a return rate of 84.1%. From these, six questionnaires were excluded from the analysis as they had been filled out repeatedly or took an unusually long time to complete. Additionally, ten questionnaires were excluded because the respondents chose the same answer for all questions on the survey. Consequently, 434 valid questionnaires were utilized for data analysis, yielding a reasonable response rate of 81.1%. Among the 434 useful questionnaires, 226 respondents (52.07%) identified as male, while 208 (47.93%) identified as female. Regarding urban residency, 56 respondents (12.9%) indicated non-urban status, whereas 378 (87.1%) indicated urban status. As for parental education, 214 (49.31%) reported having a bachelor’s degree, and 73 (16.82%) reported having a master’s degree. In terms of the frequency of playing online games outside of class, the highest number of respondents (259, 59.68%) selected "3–4 days a week," while the lowest number (8, 1.84%) indicated "never." Furthermore, the majority of respondents (358, 82.5%) reported owning a tablet device for classroom use. The predominant age group of the interviewed students was 18–22 years old (95.6%).

### 3.2. Measures

This study employs the scales that are widely recognized in previous research. Since the study was conducted in Thailand, the original English version of the questionnaire underwent translation into Thai following the methodology established by the translation committee. This was done to ensure the closest possible equivalence between the translated scale and the original scale. The questionnaire was revised by three Chinese researchers specializing in Thai language disciplines, along with local Thai researchers. Revisions involved clarifying certain words in multiple items and adjusting the questionnaire format to guarantee the alignment of meaning between the English and Thai versions. This study employed a cross-sectional quantitative approach, which allowed for the simultaneous investigation of various variables, including digital educational games, student motivation, student engagement perceptions, and the school’s digital educational environment. Responses to the study variables were scored using a 5-point Likert scale, ranging from "1" to "5," indicating the level of agreement with the item descriptions, varying from high to low.

Digital educational games: The quantitative data analysis was employed to answer the research questions using this measure, while the qualitative data provided a detailed description of the practical aspects of digital educational games from the learners’ perspective (Creswell & Plano Clark, 2006) [51]. The project assessed students’ perceptions regarding digital education games by evaluating their knowledge, support, and perceived importance of digital education. The Cronbach’s alpha coefficient for reliability is 0.893.

Students’ motivation for learning: The "PIAAC Adult Literacy Survey Technical Report" published by OECD presents Chapter 18, "Quantitative Results," which includes the outcomes of the survey regarding "willingness to learn." This chapter contains statistical tables with survey results on participants’ willingness to learn, particularly in digital educational games [52]. For instance, an item might state, "I strongly desire to learn and master knowledge and skills through digital educational games." The Cronbach’s alpha coefficient for reliability is 0.794.

Student learning engagement: To measure student learning engagement, the researchers utilized the Online Learning Technology Acceptance Scale derived from the Technology Acceptance Scale proposed by Davis (1985) [53]. This scale comprises four dimensions: perceived usefulness, perceived ease of use, attitude toward service, and willingness to continue to use, with responses scored on a 5-point Likert scale [54]. An example statement from this scale is, "Whenever I learn something new, I associate it with what I have learned in previous digital educational games and gain a deeper understanding of the new knowledge." The Cronbach alpha for this measure is 0.910.

Digital environment: The learning environment evaluation relied on the widely used instrument known as the Dundee Ready Education Environment Measure (DREEM) [54]. The "Digital environment" encompasses the fundamental conditions for digital learning, including hardware environment, software platform, and digital resources. For instance, a statement from the evaluation is "Our school’s technology services department with related responsibilities is clearly defined." The Cronbach alpha value for this measure is 0.842.

Control variables: To account for potential complex effects, several control variables were selected, including gender, household registration, parental education, ownership of tablet devices, and frequency of online gaming. Gender, age, and household registration were control variables due to their positive predictive relationship with students’ learning engagement [55]. Parental education served as a control variable since families significantly influence students’ beliefs about digital media, and parental education plays a pivotal role in students’ use of technology for learning. It positively predicts the school’s digital education environment, students’ motivation to learn, and digital device availability, making it a relevant control variable for this study [56]. Lastly, the possession of tablet devices and engagement in online games were used as control variables because they reflect differences in students’ learning style preferences and positively predict the impact of digital educational games on students’ motivation to learn [57].

## 4. Results

### 4.1. Confirmatory factor analysis

We employed Mplus 8.3 (Muthén & Muthén, 2017) to evaluate the discriminant validity of the variables through a series of validated Confirmatory Factor Analyses (CFA) in our study [58]. The outcomes of the CFA are displayed in Table 1. As mentioned earlier, the hypothesized four-factor model, comprising digital educational games, student learning engagement, the school’s digital environment, and students’ motivation for learning, demonstrated satisfactory fit across all indicators (χ2 = 3889.424, df = 3074, CFI = 0.944, TLI = 0.943, SRMR = 0.051, RMSEA = 0.035). Furthermore, a comparison of the hypothesized model with all alternative models using chi-square split tests revealed that our proposed model exhibited the most favorable fit to the data, supporting the discriminant validity of the measurements.

**Table 1:** The results of confirmatory factor analyses.

Measurement model	χ^2^	df	Δχ^2^	CFI	TLI	SRMR	RMSEA
The hypothesized four-factor model	3889.424	3074		0.944	0.943	0.051	0.035
Three-factor model (combining DEG and DE)	5633.681	3077	1744.257***	0.825	0.820	0.091	0.062
Three-factor model (combining DE and ML)	5860.626	3077	1971.202***	0.809	0.804	0.095	0.065
Two-factor model (combining DEG and SLE; DE and ML)	7818.954	3079	3929.530***	0.675	0.667	0.138	0.084
One-factor model (combining DEG, SLE, DE and ML)	10259.192	3080	6369.768***	0.508	0.495	0.141	0.104

Note: CFI = Comparative Fit Index; TLI = Tucker-Lewis index; SRMR = Standardized Root Mean Square Residual; RMSEA = Root Mean Square of Approximation;

Digital Educational Games = DEG; Student Learning Engagement = SLE; Digital Environment = DE; Motivation for Learning = ML

### 4.2. Descriptive statistics and correlations

SPSS 25.0 was employed to compute the mean and standard deviation of the study variables, while Pearson’s correlation was used to determine the correlation coefficients between each variable. The outcomes are presented in Table 2. The correlation analysis data indicate the following: (1) Each variable’s mean and standard deviation showed no abnormalities. (2) Table 2 displays the means, standard deviations, and correlation coefficients, revealing a significant positive correlation between digital educational games and students’ motivation for learning (r = 0.430, p < 0.01), which provides preliminary support for H1. Additionally, digital educational games exhibited a positive correlation with student learning engagement (r = 0.386, p < 0.01), and students’ perceptions of classroom engagement displayed a positive correlation with their motivation for learning (r = 0.461, p < 0.01). These results satisfy the conditions necessary to test for a mediating role.

**Table 2:** Means, standard deviations, correlations.

Variables	Mean	SD	1	2	3	4	5	6	7
1. City	1.479	0.500							
2. Education	1.649	0.477	-0.069						
3. Network equipment	3.601	1.072	0.069	0.214**					
4. Frequency of playing online games	1.023	0.150	0.037	-0.080	0.115*				
5. Digital educational games	3.222	0.884	0.013	0.007	0.008	0.102*			
6. Student learning engagement	3.329	0.928	0.049	-0.019	0.059	0.013	0.386**		
7. Digital environment	3.333	0.948	0.010	0.007	-0.010	0.096*	0.465**	0.468**	
8. Motivation for learning	3.316	0.961	-0.019	-0.029	-0.018	0.135**	0.430**	0.461**	0.498**

Notes. N = 434. SD: standard deviations.

*: p < 0.05;

**: p < 0.01;

***: p < 0.001

(Control variable: City = Whether or not you have an urban household; Education = Parents’ education; Network equipment = Possession of digital equipment)

### 4.3. Hypothesis testing

In this study, we conducted a regression analysis to examine the relationship between digital educational games and students’ motivation for learning. The data were analyzed, and the results are presented in Table 3. We used several control variables, including whether the interviewed students have urban household registration, their parent’s education level, the frequency of participating in online games. Digital education games were treated as the independent variable, while students’ motivation was the dependent variable. The analysis revealed that digital education games significantly and positively impacted student motivation (Model 1, B = 0.458, p<0.001), thereby supporting Hypothesis 1.

**Table 3:** Regression results for direct effect model and mediation model.

Variables	Model 1	Model 2	Model 3	Model 4
	X→Y	X→M	M→Y	X→M→Y
Constant	1.431***	1.993***	1.125**	0.700*
City	-0.057	0.072	-0.087	-0.084
Education	-0.053	-0.065	-0.016	-0.030
Network equipment	-0.003	0.050	-0.023	-0.021
Frequency of playing online games	0.580*	-0.150	0.813**	0.635*
Digital educational games	0.458***	0.407***		0.309***
Student Learn Engagement			0.480***	0.366***
R^2^	0.195***	0.155***	0.233***	0.300***
F	20.702***	15.748***	25.943***	30.566***

Notes. N = 434.

*: p < 0.05;

**: p < 0.01;

***: p < 0.001

(Control variable: City = Whether or not you have an urban household; Education = Parents’ education; Network equipment = Possession of digital equipment)

Based on the results presented in Table 3 (Model 2), we examined the relationship between digital education and student learning engagement. After controlling for the corresponding variables, the data demonstrated that digital educational games significantly and positively impact student learning engagement in the classroom (b = 0.407, p < 0.001). This finding suggests that integrating digital educational games in schools enhances students’ perception of active participation and engagement in learning. Thus, Hypothesis 2 is supported.

The results obtained from Model 3 indicate a significant and positive influence of student learning engagement on their motivation for learning (b = 0.480, p<0.001). This suggests that when students perceive themselves as actively participating in their educational experiences, their motivation to learn is higher. Hence, we find strong support for hypothesis 3.

In Model 4, where students’ motivation for learning is the dependent variable, student learning engagement and digital educational games are included as independent variables. The results show that student learning engagement continues to significantly and positively affect students’ motivation for learning (b = 0.366, p < 0.001). The stepwise test of the mediation effect reveals that the positive impact of digital educational games on students’ motivation for learning weakens after the inclusion of student learning engagement (b = 0.309, p < 0.001), leading to an improvement in the model’s fit (R^2^ = 0.300). Based on these data, it can be tentatively concluded that student learning engagement mediates between digital educational games and students’ motivation for learning.

### 4.4. Mediation effect test

The mediating effect of student learning engagement was further examined through a Bootstrap test using the Process_v4.5 program developed by Hayes (2012) within the SPSS software [59]. The results in Table 4 confirm a partial mediating effect of student learning engagement between digital educational games and students’ motivation for learning. The indirect impact of student learning engagement is 0.1491, accounting for 32.58% of the total impact, with a confidence interval of (0.1055, 0.1987), which excludes 0. Moreover, the absolute and direct effects of digital educational games on students’ motivation for learning are both 0.4577, with confidence intervals of (0.1055, 0.1987) not encompassing 0. This indicates that students’ learning engagement indeed mediates the relationship between digital educational games and students’ motivation for learning, supporting the validity of hypothesis 4.

**Table 4:** Bootstrap test of mediating effect.

Path	Effect Value	BootSE	BootLLCI	BootULCI	Effectiveness Ratio
Total effect	0.4577	0.0474	0.3646	0.5508	
Direct effects	0.3086	0.0479	0.2109	0.3985	0.6742
Indirect effects	0.1491	0.0239	0.1055	0.1987	0.3258

Notes. N = 434. Unstandardized regression coefficients are reported. Bootstrap sample size 5,000. LL = lower limit; UL = upper limit; CI = confidence interval.

In line with hypotheses 5 and 6 in this study, the digital environment moderates the relationship between digital educational games and student learning engagement, as well as the relationship between student learning engagement and motivation for learning. The results from the moderation model, presented in Table 5, support hypotheses 5 and 6 by revealing a significant interaction effect between digital educational games and the digital environment on student learning engagement (B = 0.205, SE = 0.050, p < 0.001), as well as between student learning engagement and the digital environment on motivation for learning (B = 0.263, SE = 0.047, p < 0.001).

**Table 5:** Regression results for moderating effect.

Predictor	Student Learning Engagement		Motivation for learning	
	B	SE	B	SE
Moderation model				
Constant	1.592***	0.367	0.761*	0.360
City	0.067	0.076	-0.068	0.074
Education	-0.079	0.082	-0.041	0.080
Network equipment	0.056	0.036	-0.015	0.036
Frequency of playing online games	-0.316	0.256	0.660	0.249
Digital educational games	0.260***	0.049		
Student learning engagement			0.284***	0.045
Digital environment	0.350***	0.046	0.315***	0.046
Digital educational games ×Digital environment	0.205***	0.050		
Student learning engagement ×Digital environment			0.263***	0.047

Notes. N = 434.

*: p < 0.05;

**: p < 0.01;

***: p < 0.001

(Control variable: City = Whether or not you have an urban household; Education = Parents’ education; Network equipment = Possession of digital equipment)

**Fig 2:**
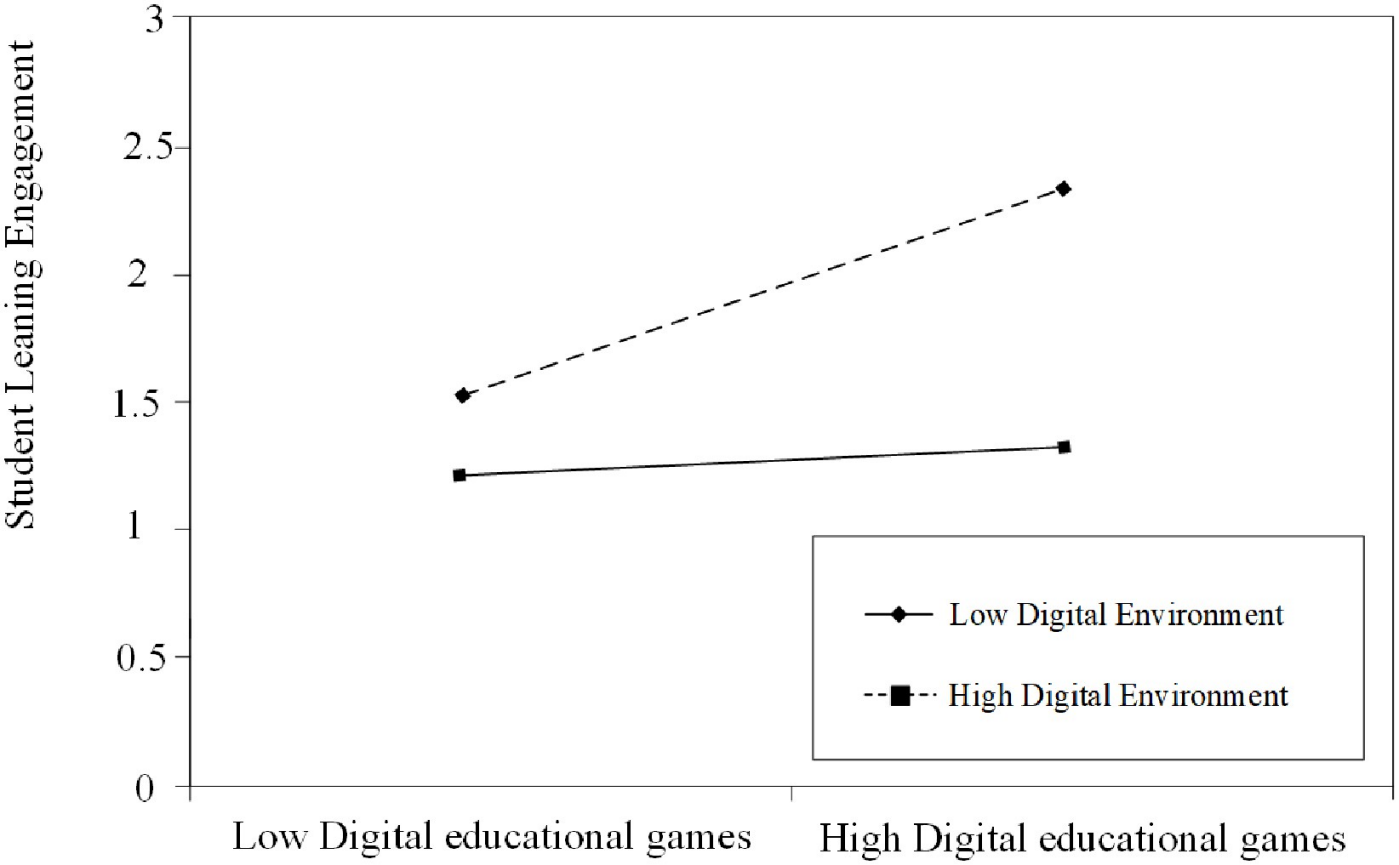
The moderating role of digital environment on the relationship between digital educational games and student learning engagement.

**Fig 3:**
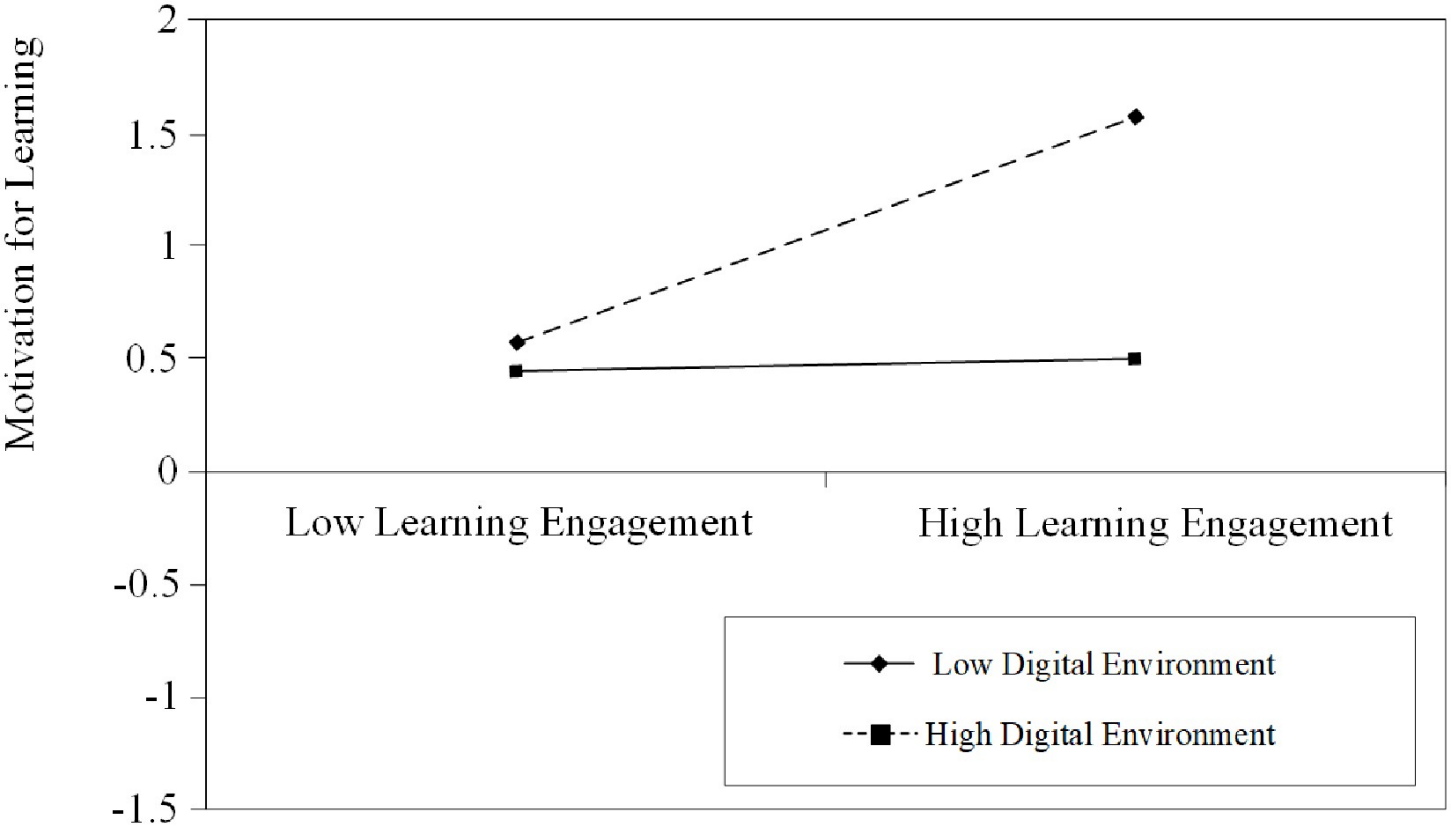
The moderating role of digital environment on the relationship between student learning engagement and motivation for learning.

The moderating effect of the digital environment (mean ± 1 SD) was visually analyzed using slope plots to observe the impact of digital educational games on student learning engagement in various school digital environments and the effect of student learning engagement on student motivation through different school digital environments. Fig 2 shows that the positive relationship between digital educational games and students’ learning engagement is stronger in high-level school digital environments. This means that in schools with advanced digital environments, digital educational games significantly impact students’ learning engagement. Conversely, in schools with low-level digital settings, the impact of digital educational games on students’ learning engagement is weaker. The same trend is observed in Fig 3, where higher levels of the school’s digital environment are associated with a stronger positive correlation between student engagement and motivation. These findings further support Hypotheses 5 and 6.

To investigate the moderated mediation model (H7), we analyzed the conditional indirect effects of digital educational games on students’ motivation for learning through student learning engagement at three different levels of the digital environment: one standard deviation above the mean, the mean, and one standard deviation below the mean of the school’s digital infrastructure. As presented in Table 6, the results indicate that at lower levels of digital environments, the indirect effect of perceived student learning engagement was not significant (Effect = 0.0073, 95% CI [-0.0504, 0.0617]), with a confidence interval that includes 0. However, at higher levels of digital environments, the indirect effect of perceived student learning engagement was highly significant (Effect = 0.1466, 95% CI [0.0964, 0.2021]). This suggests that the impact of digital educational games on students’ motivation for learning increases as the level of the digital environment improves. Furthermore, the confidence interval for the difference between higher and lower levels of the digital environment was [0.0691, 0.2225], indicating a significant moderating mediating effect. These findings provide robust support for Hypothesis 7.

**Table 6:** Regression results for moderated mediation model.

Moderated mediation model	Effect Value	BootSE	BootLLCI	BootULCI
Low Digital environment (Mean − 1 SD)	0.0073	0.0282	-0.0504	0.0617
Digital environment (Mean)	0.0770	0.0194	0.0400	0.1177
High Digital environment (Mean + 1 SD)	0.1466	0.0270	0.0964	0.2021
Difference	0.1393	0.0388	0.0691	0.225

Notes. N = 434. Unstandardized regression coefficients are reported. Bootstrap sample size 5,000. LL = lower limit; UL = upper limit; CI = confidence interval.

## 5. Discussion

This study investigates the relationship between digital educational games and students’ motivation for learning, drawing upon behaviourist and social cognition theories. Moreover, it examines the potential mediating role of student learning engagement and the moderating role of the school’s digital environment in this context.

The findings of this research reveal a positive association between digital educational games and students’ motivation for learning. Furthermore, the study observed that student learning engagement partially mediates the relationship between digital educational games and students’ motivation for learning. In other words, when students actively engage with digital educational games, it enhances their motivation to learn [60].

Additionally, the school’s digital environment plays a significant moderating role in the link between digital educational games and student learning engagement, as well as the connection between students’ learning engagement and their motivation for learning. In essence, the quality and resources provided by the school’s digital environment can influence the effectiveness of digital educational games in engaging students and ultimately impact their motivation levels [61].

Notably, the school’s digital environment also exerts a crucial mediating effect on the relationship between digital educational games and students’ motivation for learning through student learning engagement. In other words, the digital environment provided by the school can either enhance or hinder the impact of digital educational games on students’ motivation levels, depending on how well it supports and complements the learning experiences offered through these games.

In conclusion, this study sheds light on the importance of digital educational games in fostering students’ motivation for learning. By understanding the mediating and moderating factors involved, educators and policymakers can effectively utilize digital educational games to enhance student engagement and motivation, taking full advantage of the school’s digital environment to create a conducive and enriching learning atmosphere [62].

### 5.1. Theoretical contributions

First, we investigated the impact of digital educational games on students’ motivation for learning, drawing on the behaviorist theory. While digital education, including the use of digital educational games, has garnered considerable attention in recent research, there remains a need for a more comprehensive exploration of their effects on student learning [63]. In line with the existing literature emphasizing the influence of digital educational games, our study reveals that students exposed to such games exhibited higher motivation to learn. Moreover, we extend the application of behaviorist theory by considering digital educational games as ’stimuli’ that can positively influence students’ motivation for learning.

Furthermore, our findings highlight the vital role of student learning engagement in mediating the relationship between digital educational games and students’ motivation for learning. Consistent with previous research, we demonstrate that student learning engagement is pivotal in shaping students’ connection with digital educational games. This observation provides support for both Behaviorist theory and Social cognition theory.

Third, we identify the school’s digital environment as a crucial factor influencing how digital educational games impact students’ motivation for learning. This finding aligns with prior research that underscores the significant role of school digital environments in shaping students’ learning experiences. Additionally, we extend social cognition theory by revealing how the school’s digital environment, as an environmental intervention, interacts with digital educational games to influence student learning engagement (individual behavior) and subsequently impacts students’ motivation for learning (individual behavior) [64–66]. This addresses the necessity to focus on perceptual effects on students’ learning development.

In summary, this study contributes to understanding the relationship between digital educational games and students’ motivation for learning, supported by behaviorist theory and social cognition theory. By recognizing the mediating role of student learning engagement and the moderating influence of the school’s digital environment, educators and policymakers can harness the potential of digital educational games to foster students’ motivation and create a conducive learning environment for their overall development.

### 5.2. Practical implications

This study carries significant practical implications, with two main findings of note. The empirical evidence establishes a positive relationship between digital educational games and students’ motivation, mediated through their learning engagement. Consequently, as Oraif and Elyas (2021) suggest, it becomes imperative for schools to transition from traditional teacher-centered lecturing approaches to adopting digital educational games as an innovative pedagogical strategy [67]. By incorporating these games into teaching methods, students can focus on their abilities and observe their peers’ engagement, fostering interest and active participation. This, in turn, enhances their knowledge and skills.

To facilitate this transition, especially within the universities that are notably more open and flexible, should prioritize ensuring that digital educational games align with students’ preferences, thus encouraging their active involvement in the learning process [68].

Secondly, the role of the school’s digital environment is pivotal in strengthening the impact of digital educational games on students’ motivation for learning. Recognizing the advantages of a well-developed digital environment in schools, management should continue investing in and fortifying the digital infrastructure of educational institutions. Equipping students with suitable digital devices will aid their adaptation to the digital educational games’ teaching mode and enhance their sense of participation.

Furthermore, According to Falloon (2020), schools should prioritize enhancing teachers’ digital literacy to integrate digital educational games into their teaching methodologies effectively [41]. Bozkurt et al. (2020) suggest that by taking proactive steps, such as increased financial support from the government for schools to acquire network equipment and introduce digital educational games, can further boost students’ motivation to learn [69].

The government can also offer support by focusing on information technology development, including network infrastructure, and implementing talent introduction policies to attract digitally proficient individuals who can contribute to the educational environment.

Finally, It aligns with Vlachopoulos’s perspective that a high-level school digital environment enhances students’ engagement in digital educational games and bolsters their motivation to learn. This, in turn, diminishes reliance on online games, fostering a constructive and collaborative relationship among schools, parents, and students [70].

In conclusion, the study underscores the importance of incorporating digital educational games into the educational landscape. It highlights the crucial role of the school’s digital environment in promoting students’ motivation for learning. By embracing these findings and taking proactive measures, schools and governments can enrich the educational experience, encouraging students’ active participation and overall academic success.

### 5.3. Limitations and future directions

Several limitations of this study warrant acknowledgement. Firstly, the homogeneity of the data source used in our research could have helped the ability to track the sustainability and duration of student motivation. Consequently, causal interpretations are constrained. To address this limitation, future research should consider employing a longitudinal research design to monitor long-term changes in student learning motivation and explore potential predictors over time.

Secondly, the application of digital educational games in daily teaching activities within secondary schools in Thailand is currently restricted due to specific requirements concerning the school’s network infrastructure and students’ home conditions. This restriction resulted in a small sample size for this study, limited to three universities, thus compromising the generalizability of the findings. To enhance the external validity of the results, future research should expand the sample size to encompass a more diverse set of schools. Additionally, to glean further insights into the impact of digital educational games on student motivation, future studies should differentiate between various grade levels. Different grade levels may yield distinct findings from varied exposure and ongoing cognitive development. Therefore, investigating a broader range of study groups, including public and private universities or higher and lower grades, would facilitate a comprehensive examination of the effects of digital educational games on students’ motivation to learn.

Thirdly, this study solely explored the moderating effects of the school’s digital environment on students’ motivation. However, according to social cognition theory, numerous situational factors can influence student learning. Consequently, future research should delve into the mechanisms through which other contextual factors influence the impact of digital educational games on students’ motivation to learn, particularly by examining their perceptions of engagement. This may involve investigating variables such as the task difficulty of digital educational games or teachers’ digital literacy as potential mediating factors.

In conclusion, this study has illuminated valuable insights into the relationship between digital educational games and student motivation for learning. Nevertheless, it is essential to recognize the limitations outlined above to facilitate more robust and comprehensive research in the future. Addressing these limitations will not only strengthen the validity and generalizability of findings but also contribute to a more nuanced understanding of the role of digital educational games in shaping students’ motivation and learning outcomes.

## 6. Conclusions

This study seeks to illuminate the relationship between digital educational games and students’ motivation for learning. In doing so, it offers valuable insights and theoretical underpinnings for a fresh approach to understanding student development and advancing scholarly research.

Our findings are rooted in the tenets of two central academic theories—the behaviorist learning theory and the contextual cognitive theory. These theories act as the pillars supporting our research framework. A significant point of emphasis in our findings underscore digital educational games can stimulate students’ motivation for learning, which is mediated by student learning engagement. By augmenting their engagement, fostering a positive attitude towards learning.

Furthermore, our research indicates that a high digital environment in schools also exerts a moderating influence on this process. Within a conducive digital setting, students are more inclined to be drawn into digital educational games, which, in turn, amplifies their zeal motivation for learning.

While our study has certain limitations, we are optimistic that it will serve as a catalyst for other scholars to delve deeper into the domain of digital education. Through rigorous research, We can better explore the application of digital education in the learning process of students.

## Supporting information

S1 Data (XLSX)
